# Molecular Epidemiology of Beak and Feather Disease Virus (BFDV), Avian Polyomavirus (APV-1), Psittacid Herpesvirus 1 (PsHV-1), and Avian Metapneumovirus (aMPV) in Birds Kept as Non-Traditional Companion Animals (NTCAs) in Italy

**DOI:** 10.3390/ani15152164

**Published:** 2025-07-22

**Authors:** Riccardo Baston, Claudia Maria Tucciarone, Alberto Caudullo, Francesca Poletto, Matteo Legnardi, Mattia Cecchinato, Michele Drigo, Giovanni Franzo, Diego Cattarossi

**Affiliations:** 1 Department of Animal Medicine, Production and Health (MAPS), Padua University, 35020 Legnaro, PD, Italy; 2 Casale sul Sile Veterinary Clinic, 31032 Casale sul Sile, TV, Italy; 3 Tropicarium Park, 30016 Jesolo, VE, Italy

**Keywords:** BFDV, APV-1, PsHV-1, aMPV, molecular biology, Italy, non-traditional companion animal (NTCA)

## Abstract

The non-traditional companion animal (NTCA) sector, particularly involving avian species, has significantly expanded in Italy, raising concerns over the spread of infectious diseases. These animals can harbor various pathogens and act as reservoirs, posing risks to native wildlife through legal or illegal trade, escapes, or intentional releases. However, the epidemiology of avian pathogens in NTCAs remains poorly understood and is typically investigated only in symptomatic individuals. In the present study, cloacal and choanal cleft swabs were collected from 319 ornamental and raptor birds across 19 families, pooled and tested for beak and feather disease virus (BFDV), avian polyomavirus (APV-1), psittacid herpesvirus 1 (PsHV-1), and avian metapneumovirus (aMPV). BFDV and APV-1 were detected in 13.79% and 2.19% of birds, respectively, with five co-infections. No cases of PsHV-1 or aMPV were found. Both viruses showed a higher prevalence than in previous Italian and most of international studies, with several non-psittacine species, including birds of prey, testing positive—some for the first time. Mixed-species settings and participation in public exhibitions were proven as significant infection risk factors. The study highlights the growing relevance of BFDV and APV-1 in non-commercial birds and recommends improved biosecurity and preventive screening to reduce disease spread and safeguard animal health.

## Simple Summary

This study investigates the circulation of beak and feather disease virus (BFDV), avian polyomavirus (APV-1), psittacid herpesvirus 1 (PsHV-1), and avian metapneumovirus (aMPV) in Italy, focusing on non-traditional companion animals (NTCAs), including both clinically affected and healthy birds. The main objectives were to assess pathogen prevalence, host tropism, co-infection patterns, and potential risk factors. While PsHV-1 and aMPV were not detected, BFDV and APV-1 were identified in 13.79% and 2.19% of tested birds, respectively. Co-infection was observed in five individuals. These findings indicate a higher prevalence than previously reported in Italy and in most other countries. Participation in public exhibitions and housing in mixed-species settings emerged as significant risk factors for infection. Furthermore, several bird species not previously associated with these viruses tested positive, expanding their known host range. Given the growing popularity of NTCAs and the increasing frequency of interactions between wild and domestic birds, these results underscore the need to strengthen biosecurity in aviculture and pet trade contexts. In particular, screening protocols before public bird gatherings and the implementation of preventive health measures are recommended to reduce the risk of viral transmission and safeguard both animal health and biodiversity.

## 1. Introduction

The sector of non-traditional companion animals (NTCAs) has significantly expanded in Italy over the past decades, with birds playing a major role both in terms of numbers and variety of the species being bred and commercialized (ASSALCO Report, 2025; https://www.assalco.it/). While exotic species such as canaries, true finches, and parakeets represent a significant portion of the market, a growing and non-negligible trend involves animals previously classified as farm animals, including chickens, ducks, and other poultry, which are now increasingly adopted as companion animals. Additionally, falconry has a long history and the use of birds of prey as working animals is a tradition that continues today [1]. However, their role has evolved, as they are now increasingly kept by owners for aesthetic purposes, exhibitions, competitions, and companionship. Furthermore, the compassionate rescue and care of injured synanthropic animals, such as pigeons, may contribute to the diversification of companion animal species.

The increase and heterogeneity of animal populations, the consequent trade and exchanges come with a greater risk of pathogen introduction and circulation [2,3,4,5,6]. Moreover, the inclusion of farm animals as companion species should be considered a potential source of introduction of previously known pathogens into a new environment. Similar concerns apply to birds of prey, which, due to their natural behaviour and activities, may act as an interface between wild and domestic species, facilitating pathogen transmission [7,8,9].

While the implications of such complex interactions have been at least partially explored—particularly in the context of zoonotic diseases, especially bacterial ones—our understanding of viral pathogens remains more limited. In Italy, in particular, most available studies have focused on a restricted number of viruses, typically chosen based on prior knowledge of their host tropism and primarily targeting animals exhibiting compatible clinical signs [10,11]. Consequently, our understanding of viral circulation is largely shaped by stringent inclusion criteria, both in terms of the studied pathogens and the considered host species.

Beak and feather disease virus (BFDV), avian polyomavirus 1 (APV-1), and psittacid herpesvirus 1 (PsHV-1) are among the most significant viral pathogens affecting companion birds, particularly psittacines, due to their high transmissibility and severe clinical impact.

BFDV, species *Circovirus parrot*, belongs to the family *Circoviridae*, genus *Circovirus*, and is characterized by a single-stranded circular DNA genome (ssDNA) of approximately 2 kb. It has two major open reading frames (ORFs), ORF1 and ORF2, encoding the viral replication-associated protein (Rep) and the major structural capsid protein (CP), respectively [6,12]. BFDV is the causative agent of psittacine beak and feather disease (PBFD), an often-fatal illness that may follow a peracute, acute, or chronic course in several psittacine species. Infected birds commonly exhibit depression, lethargy, anaemia, and abnormal feather growth, with some species also developing beak and claw deformities [13]. The immunosuppression induced by BFDV frequently leads to secondary infections and death. The virus is highly contagious, through both vertical and horizontal transmission, particularly in crowded captive environments such as breeding facilities, where environmental and fomite contamination plays a significant role in pathogen spread [10].

Similarly, aves polyomavirus 1 (APV-1), species *Gammapolyomavirus avis*, is a highly contagious virus affecting psittacine birds, but it has also been detected in *Passeriformes* and *Falconiformes*. APV-1 belongs to the family *Polyomaviridae*, genus *Polyomavirus*, and has a double-stranded circular DNA genome of approximately 5kb. It causes a pathology known as budgerigar fledgling disease (BFD), commonly associated with acute death and clinical signs such as abdominal distension, abnormal feathering, and haemorrhages in subcutaneous and subserosal regions, which mostly affects birds of the *Psittaciformes*, and particularly budgerigars [13]. Originally isolated in North America, APV-1 has since been reported in several countries worldwide, with European and Asian surveys highlighting its global spread [14,15].

Another critical pathogen in companion birds is psittacine herpesvirus 1 (PsHV-1), species *Iltovirus psittacidalpha1*, responsible for Pacheco’s disease and other herpesvirus-associated conditions. PsHV-1 belongs to the family *Herpesviridae*, subfamily *Alphaherpesvirinae*, and is characterized by a double-stranded DNA genome that enables it to establish latent infections. PsHV-1 infection often leads to severe systemic disease, especially in naïve or stressed birds, causing sudden death, hepatitis, splenomegaly, and haemorrhagic lesions. Clinical signs may also include depression, anorexia, greenish urates, and neurological manifestations [16]. Transmission occurs through direct contact with infected birds, aerosols, and contaminated food or water sources. A major concern is that PsHV-1 infections can remain latent, with asymptomatic carriers acting as reservoirs for viral spread. Stressful conditions, such as transportation, overcrowding, or concurrent infections, can trigger viral reactivation and lead to disease outbreaks. This is particularly relevant in breeding and aviary settings, where transmission risks are high [17]. Given the high mortality rate associated with PsHV-1 outbreaks, early diagnosis through PCR and serological testing is crucial for disease control and management [18].

Avian metapneumovirus (aMPV), species *Metapneumovirus avis*, is a single-stranded, non-segmented, negative-sense RNA virus of approximately 13.3–14 kb, belonging to the family *Pneumoviridae*. It is a highly infectious pathogen primarily affecting turkeys, ducks, and chickens, although it may potentially infect other avian species. aMPV causes upper respiratory infections, predisposing birds to opportunistic bacterial pathogens, which can lead to severe respiratory disease, high morbidity, and mortality. Additionally, it can affect the reproductive system, leading to reduced egg production and quality [19].

aMPV has long been recognized as a cause of economic losses in the poultry industry, with its significance increasing in recent years [19]. The first identified subtype (aMPV-A) emerged in South Africa in the 1970s, followed by the spread of another subtype, aMPV-B, in Europe [20]. In 1996, aMPV-C was first detected in North America, primarily affecting turkeys and wild birds, while aMPV-D was retrospectively identified in French samples from the 1980s [19]. However, recent surveillance has reported new subtypes in North American black-backed gulls [21] and monk parakeet chicks [22], while aMPV-B and aMPV-A have recently emerged in the U.S. [23], posing new diagnostic and surveillance challenges. More generally, aMPV has revealed a greater host flexibility than expected, with an overlap among all host categories (poultry, companion, and wild birds), making its investigation worthy [24].

The present study investigates the circulation of a diverse set of viral pathogens relevant to companion, poultry, and wild bird populations, testing various species of unconventional companion birds, including both clinically affected and healthy individuals. The main aim was to assess the frequency, host tropism, interactions, and risk factors for infection, contributing to a more comprehensive understanding of the epidemiological landscape in the most encountered birds in veterinary practices.

## 2. Materials and Methods

### 2.1. Sample Collection

The samples analysed in this study were collected at the Casale sul Sile Veterinary Clinic (Casale sul Sile, Italy) or during clinical activities conducted at other facilities by the responsible veterinarian, an NTCA specialist. Samples were obtained from ornamental birds and birds of prey presented for routine health check-ups or due to clinical symptoms. For all subjects, information on animal species, age (categorized as young, adult, and elder, based on species-specific criteria), sex, geographical origin, attendance to exposition/fair, and clinical sign were recorded. Animal owners received information about the study aim, sampling procedures, and confidentiality of the collected data and provided written informed consent prior to sample collection. Sampling was performed in accordance with applicable ethical regulations and guidelines for animal welfare (University of Padua O.P.B.A. approval certificate n.106432).

**Figure 1:**
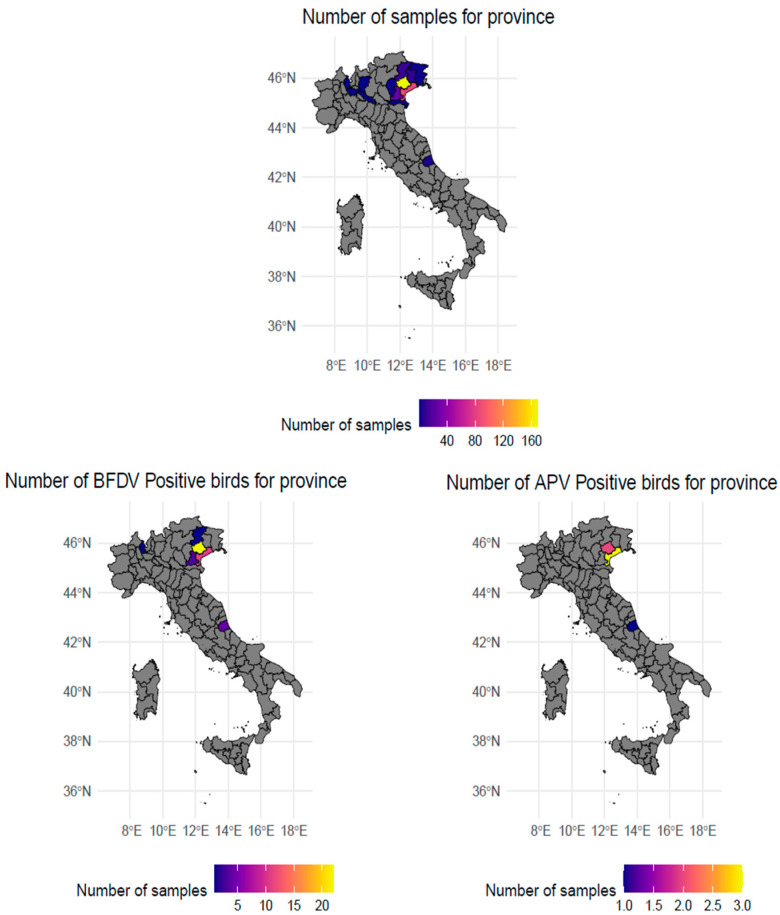
Map of Italy depicting the provinces where the sample originated from (upper figure). The number of samples positive to BFDV and APV-1 are depicted in the lower left and right panel, respectively.

Since the clinic where samples were collected serves as a reference centre for NTCAs, it receives cases and patients from a broad geographical area, including Lombardy, Veneto, Friuli Venezia Giulia, and some samples from Teramo province (Figure 1).

Subjects were included in the study regardless of the species or presence of specific clinical signs, with the aim of obtaining a comprehensive and representative overview of the ornamental bird population in the region and, consequently, of the viral circulation. From each bird, both cloacal and choanal cleft swabs were collected and processed separately. In a limited number of cases, dual sampling was not feasible, or the swabs were stored in the same container; in such instances, they were considered a single sample.

Swabs were initially stored at −20 °C for a short period before transfer to the Department of Animal Medicine, Production, and Health (MAPS) at the University of Padua, where they were maintained at −80 °C until further processing.

### 2.2. Sample Processing and Pooling

Nucleic acid extraction was performed using the Viral DNA/RNA kit (A&A Biotechnology, Gdynia, Poland) following the manufacturer’s protocol. Prior to extraction, samples were resuspended in 1 mL of 1X PBS by vortexing. To optimize processing efficiency and reduce the costs without affecting sensitivity, samples were pooled in groups of five before extraction. Individual samples from pools that tested positive were subsequently extracted separately using the same kit and reanalysed.

### 2.3. Molecular Analyses

Multiplex quantitative PCR (qPCR) assays were validated for the detection of BFDV, PsHV-1, and APV-1, using the primers and probes suggested by Gibson et al. [18]. To this end, a standard curve was generated on a plasmid containing the target sequences of all viruses, which was chemically synthesized and cloned by GenScript (Rijswijk, Netherlands). The precise amount of plasmid provided by the manufacturer allowed for the estimation of the copy number, facilitating quantification. To construct the standard curve, tenfold serial dilutions of the plasmid were prepared and used to assess the performance of the assay, specifically evaluating the limit of detection and the reaction efficiency. Various primer and probe concentrations, along with different protocols, were tested to determine the optimal conditions ensuring the highest analytical performance.

All qPCR reactions were performed on the MyGo Pro ESR platform (Azura Genomics Inc., Raynham, MA, USA) using the TaqMan™ Fast Advanced Master Mix (ThermoFisher Scientific, Applied Biosystems, Waltham, MA, USA). Each reaction contained 5 µL of TaqMan™ Master Mix, 0.6 µM each of BFDV_Rep forward and reverse primers (ATACTTACYCTGGGCATTGT and CGCGCGACKTCCTTCAT, respectively), 0.6 µL each of APV_ORF1 forward and reverse primers (TGCCAGAGCGCGATTTAT and TCCGCATTCCCTTATTTGGA, respectively), and 0.3 µM of each of BFDV and APV_1 probes (FAM-ATCACGGCAGCAACAGCTCS-BHQ1 and HEX-ACAAGGGAAGTGCTGAGCAATCGT-BHQ1, respectively). Ultrapure water was added up to a final volume of 10 µL.

PsHV-1 was tested in independent runs using the primers UL16 (TCAACGACGTCAACGTCTG) and UL16 (GGTACAGGTATATYCTCAGYGA) and the probe (FAM-AGTCCGYAACGAYYCKCTWKTGACCATC-BHQ) at the same conditions.

The thermal cycling conditions consisted of an initial enzyme activation step at 95 °C for 2 min, followed by 45 cycles of denaturation at 95 °C for 10 s and annealing/extension at 60 °C for 20 s, with fluorescence signals acquired at the end of the extension phase. MyGo Pro ESR instrument (Azura Genomics Inc., Raynham, MA, USA) and Novacyt MyGo Pro ESR software v1.1 (Azura Genomics Inc., Raynham, MA, USA) were used for qPCR assay setup and data visualization. The analytical sensitivity was determined as 10 copies/µL for BFDV, APV-1, and PsHV-1, with estimated amplification efficiencies of 97.54%, 96.42%, and 101.50%, respectively. No false-positive results were observed when testing negative controls, tissues from SPF chickens originating from previous experimental studies, or virus samples from other host species previously characterized as positive for viruses belonging to the same genera as those included in the study.

The presence of aMPV was tested using previously validated protocols, as described in Tucciarone et al. [24], which allowed the screening for A, B, C, and recently identified gull and parakeet subtypes. The assays were performed in two separate multiplex RT-PCRs using SuperScript™ III Platinum™ One-Step qRT-PCR Kit (Invitrogen™, Thermo Fisher Scientific Inc., Waltham, MA, USA) on LightCycler^®^ 96 Instrument (Roche, Basel, Switzerland).

### 2.4. Statistical Analysis

The viral detection frequency and corresponding confidence intervals were estimated using the Wald method, as implemented in the epiR 2.0.84 R package [25]. Associations between categorical variables were tested using Fisher’s exact test, while continuous variables were compared using either the non-parametric Kruskal–Wallis or Mann–Whitney tests, depending on the number of levels considered. Bonferroni correction was applied for multiple comparisons. All analyses were performed in R and the significance level was set at 0.05.

## 3. Results

### 3.1. qPCR and RT-qPCR Tests

Between February and December 2024, cloacal and choanal cleft swabs were obtained thorough a convenience sampling from 319 ornamental birds and birds of prey presented for routine health check-ups or due to clinical symptoms. The sampled birds belonged to 19 families, originating from 13 Italian provinces, especially from Northern Italy (Figure 1). One additional sample originated from Slovenian birds.

Forty-four birds were positive for BFDV (13.79% [95CI: 10.44–18.01%]) and 7 (2.19% [95CI: 1.07–4.46%]) for APV-1. Five out of the total 319 birds were positive to both pathogens (1.567% [95CI: 0.671–3.616%]), revealing a significant association between the two (*p* < 0.001). The following bird families were infected by BFDV: *Accipitridae* (2/12, 16.67% [95CI: 4.70–44.80%]), *Anatidae* (1/10, 10.00% [95CI: 1.79–40.42%]), *Columbidae* (1/44, 2.27% [95CI: 0.40–11.81%]), *Estrildidae* (3/8, 37.50% [95CI: 13.68–69.43%]), *Fringillidae* (7/36, 19.44% [95CI: 9.75–35.03%]), *Phasianidae* (3/30, 10.00% [95CI: 3.46–25.62%]), *Psittacidae* (24/144, 16.67% [95CI: 11.46–23.60%]), *Strigidae* (1/7, 14.29% [95CI: 2.57–51.31%]), *Turdidae* (1/6, 16.67% [95CI: 3.01–56.35%]).

A more detailed list of involved species is provided in Table 1 and Table 2.

For APV-1, only members of *Estrildidae* (1/5, 20.00% [95CI: 3.62–62.45%]), *Phasianidae* (1/30, 3.33% [95CI: 0.59–16.67%]), and *Psittacidae* (5/144, 3.47% [95CI: 1.49–7.87%]) were found positive.

All subjects tested negative for PsHV-1 and any aMPV subtypes.

Among BFDV-positive samples, 24/44 (54.55% [95CI: 40.07–68.29%]) and 32/44 (72.73% [95CI: 58.15–83.65%]) were collected from the choanal cleft and cloaca, respectively.

In 11/44 birds (25.00% [95CI: 14.57–39.44%]), both matrices were positive, revealing significant associations in positivity detection between the two sites (*p* < 0.001).

Among APV-positive birds, 5/7 (71.43% [95CI: 35.89–91.78%]) were identified from the respiratory tract and 2/7 (28.57% [95CI: 8.22–64.11%]) from the cloaca, while none showed combined excretion.

No significant differences in BFDV viral titre excretion were observed between respiratory and enteric tract, while no statistical analysis was performed for APV-1 due to the limited number of positive samples.

### 3.2. Risk Factor Evaluation

No gender or age category effect was observed in relation to BFDV or APV-1 presence.

BFDV-positive subjects had an odds ratio of 2.56 [95CI: 1.00–7.66] for having clinical signs compared to negative ones (*p* = 0.05), while no statistically significant association was identified for APV-1 detection. The clinical signs observed in BFDV-positive animals included the following: 10 respiratory, 5 digestive, 2 reproductive, 7 haemorrhagic, 4 musculoskeletal, 5 general condition, and 3 dermatological cases. The clinical signs observed in APV-1 positive animals included the following: 2 respiratory, 1 systemic, 1 digestive, and 2 haemorrhagic cases.

The involvement of animals in fairs, exhibitions, and other public events led to an almost three-fold increase in BFDV detection risk (odds ratio = 2.93 [95CI: 1.25–6.48]; *p* = 0.009). Also in this case, no association was detected for APV-1.

## 4. Discussion

The NTCA sector has been significantly increasing in recent decades, creating an environment in which both old and new pathogens can circulate. Among the pathogens affecting psittacine birds, BFDV is probably the most studied and relevant. The present study revealed a 13.79% detection frequency in Italian birds raised as companion animals, which almost doubles the previously reported prevalence in *Psittaciformes* [10]. Although the present study included a higher variety of bird species, comparable and even higher values (~16%) were observed when considering only psittacine birds.

Although the enrolled birds did not originate from the same areas and facilities, both investigations were essentially focused on Northern Italy. Therefore, a temporal rather than spatial component in prevalence variation can be suggested. The present Italian results on BFDV are higher compared to previous reports from Costa Rica, Thailand, Chile, and Namibia but similar to or lower than those observed in captive bird populations in Australia, Thailand, and Turkey [26,27,28,29,30,31]. However, studies conducted in breeding facilities in Turkey and China showed even higher detection rates [32]. Regardless of the specific countries, among which a huge variability was observed and where geographical effects are difficult to disentangle from those related to population, diagnostic matrix, and approach, an overall trend toward an increasing detection frequency in pet shops and breeding facilities was observed. This reflects the impact of high animal densities and more frequent contact opportunities [32]. Accordingly, in the present study, birds participating in exhibitions and fairs showed almost three times higher odds of viral detection. The risk posed by these events should not be underestimated, both due to the limited biosecurity measures and the high likelihood of direct or indirect contact through feather dust and contaminated surfaces, with BFDV’s high environmental resistance allowing prolonged persistence outside the host. Preliminary testing and animal certification should therefore be considered before admission to public events. Such measures would be particularly relevant given the high percentage of animals showing no clinical signs or only unrelated manifestations, as reported in the present and previous studies.

The variety of species, including non-psittacine ones, testing positive in the present study was among the most surprising findings. While BFDV has been reported in several bird species, primarily affecting *Psittaciformes* but also some other bird families [33], such heterogeneity has rarely been reported. Among the species detected in the present study, Budgerigar (*Melopsittacus undulatus*), Cockatiel (*Nymphicus hollandicus*), Canary (*Serinus canaria*), African Grey Parrot (*Psittacus erithacus*), Blue-and-Yellow Macaw (*Ara ararauna*), Blue-Fronted Amazon (*Amazona aestiva*), White-Bellied Caique (*Pionites leucogaster*), Black-Headed Caique (*Pionites melanocephalus*), Senegal Parrot (*Poicephalus senegalus*), Sun Conure (*Aratinga solstitialis*), Rock Dove/Pigeon (*Columba livia*), European Goldfinch (*Carduelis carduelis*) and Alexandrine Parakeet (*Psittacula eupatria*) have been reported as susceptible to BFDV [26,32,34,35,36,37,38,39,40,41,42,43].

For other species in the list, such as Eurasian Scops Owl (*Otus scops*), Harris’s Hawk (*Parabuteo unicinctus*), Domestic Goose (*Anser anser* × *Anser cygnoides*), Chicken (*Gallus gallus*), Helmeted Guineafowl (*Numida meleagris*), and Common Blackbird (*Turdus merula*), there are no documented reports of BFDV infection. Of note, several of these species revealed a detection frequency comparable or even higher than the psittacine one. However, it must be stressed that the limited number of individuals sampled from most species prevents any reliable prevalence estimation.

Different factors may have contributed to the present scenario. Most samples originated from household amateur facilities rather than professionals. Non-professional owners often raise or host multiple NTCA species in the same settings, in close contact, and with limited to no biosecurity or control measures. This clearly creates optimal conditions for contact and, eventually, might enhance the risk of cross-species transmission among susceptible individuals.

Moreover, the use of a highly sensitive molecular biology diagnostic test on non-invasive sample types, such as palatine cleft or cloacal swabs—selected for ethical reasons to prevent stress and unnecessary procedures on healthy animals—does not allow the differentiation between actual infection and mere contamination (e.g., ingestion of contaminated material).

The evaluation of viral titres among host families suggested a tendency for *Psittacine* and *Phasianidae* species to display a higher shedding, which may indicate differential host tropism. However, the convenience sampling prevented any standardization of the sampling timing, and therefore variable infection and shedding phases were likely spotted, hampering consistent comparison and inference on excretion level. Nevertheless, the substantial overlap in observed BFPV titres supports the hypothesis that replication, rather than mere transitory presence, occurs in other host categories as well.

Birds of prey also were detected among the positive species, more specifically an *Otus scops* (Eurasian Scops Owl) and two *Parabuteo unicinctus* (Harris’s Hawk). An increasing number of studies has reported BFDV presence among birds of prey, from the orders *Accipitriformes* (hawks, eagles, kites), *Falconiformes* (falcons and caracaras), and *Strigiformes* (owls), sometimes at not negligible frequency [38], although the implications on animal health and species conservations in wild settings are still unknown [44,45,46,47]. In Australia, where most of these detections were reported, there is a significant ecological relationship with parrots, which is estimated to contribute to their dietary biomass [45]. In the present study, all positive samples were cloacal swabs, which could be attributable to food ingestion rather than actual viral replication. Unlike in wild animals, parrots are clearly not a dietary component of captive-raised raptors, reducing the likelihood of this hypothesis. However, psittacine birds that have escaped from captivity have widely established themselves as feral populations in Italy and have become a significant part of the local bird community, benefiting from the warmer conditions associated with climate change [48]. Therefore, contacts with these species during recreational or working hunting activities cannot be excluded.

The above-mentioned scenario further highlights the risk posed by allochthonous/invasive species. In addition to competing with native species for habitat and resources, they can contribute to the introduction and persistence of various pathogens. The broad host tropism observed in the present study—which includes species commonly residing in domestic, peri-urban, and poultry settings, like *Columbidae*, *Estrildidae*, *Fringillidae*, *Phasianidae*, etc.—represents a potential further risk.

The prevalence of APV-1 was significantly lower, both in terms of frequency and host tropism, consistent with previous Italian and international findings. However, the detection frequency, approximating ~3%, represents an increase compared to earlier reports, suggesting a dynamic epidemiological scenario. Nonetheless, study-specific variability was also observed in this case [10,28,31,49]. Co-infections between the two viruses were common, and a statistically significant association was found. Whether this reflects a common exposure risk or a positive interaction between BFDV and APV-1 remains to be determined.

The absence of herpesvirus detection might suggest limited circulation of this pathogen in Italy, as observed in other countries [50]. However, the presence of latency, the inclusion of several healthy animals in the study, and the presence of non-target species may have led to an underestimation.

Finally, no aMPV-positive subjects were identified. Although different aMPV subtypes exhibit a broad host tropism—encompassing members of the families *Anatidae*, *Columbidae*, *Falconidae*, *Psittacidae*, *Cracidae*, *Laridae*, *Rallidae*, *Corvidae*, *Ardeidae*, *Passeridae*, *Hirundinidae*, and *Sturnidae*, which align with the population enrolled in the study—the absence of positive results suggests at least an extremely low circulation in the considered settings [24]. This finding mirrors the extremely sporadic detection of aMPV in non-intensively raised species reported in previous studies.

On the contrary, prevalence is typically much higher in intensively farmed chicken populations worldwide, highlighting the strong tropism of aMPV for this host [20,51,52]. Therefore, the virus absence in the same species raised as pets could seem surprisingly. Nevertheless, the limited shedding period, low animal density, restricted contact opportunities with other individuals, absence of other predisposing factors, and relatively small sample size may all contribute to the lack of detections observed in this study. Moreover, no data on host tropism and pathogenetic features (target tissues, clinical manifestations, shedding routes, and periods) are available on the newly detected aMPV subtypes, hampering the selection of suitable time window and host species for a precise sampling, which would increase detection chances.

This study is not devoid of limitations, particularly related to sampling features, that must be considered in the result interpretation. First, the convenience-based nature of the sampling hampers the generalizability of the study and may conceal potential biases. Sampling was performed on live animals in a clinical setting, where owner consent and ethical considerations limited the use of invasive procedures. For this reason, choanal and cloacal swabs were selected to ensure a standardized and minimally invasive approach, maximizing compliance and recruitment. Although feather sampling could guarantee better performances for certain diseases, such as BFDV, and is often considered non-traumatic by veterinarians, it is perceived as invasive by many owners, particularly in the context of routine monitoring of healthy individuals. From an ethical standpoint as well, performing invasive procedures on healthy animals would have been questionable.

Furthermore, one of the aims of the study was to investigate co-infection rates. Although feather sampling may be suitable and even preferred for birds suspected of having BFDV, this matrix would have been inappropriate for the detection of pathogens associated with respiratory disease (e.g., aMPV), thereby introducing potential bias. For these reasons, and to ensure consistency across animal categories (healthy vs. diseased, and with different clinical presentations), we opted for a standardized, minimally invasive sampling strategy.

## 5. Conclusions

This study provides new insights into the epidemiology of BFDV, APV-1, and other avian pathogens in Italian non-commercial birds, highlighting a concerning trend of increasing infection prevalence, and a significant role of exhibitions and fairs in infection spreading. The broad host tropism observed, with positive detections in both psittacine and non-psittacine species, raises questions about the role of different avian taxa in viral maintenance and transmission. Additionally, the detection of BFDV in birds of prey, although possibly linked to contamination due to infected meat ingestion rather than active viral replication, warrants further investigation to assess potential implications for wildlife health and conservation. The increasing prevalence of APV-1, though still lower than BFDV, indicates the need for continued surveillance, especially given the significant rate of co-infection between these two viruses. On the other hand, the absence of herpesvirus and aMPV detection might suggest a limited circulation in the studied population, yet factors such as latency, low shedding, sampling time, or sample size constraints may have influenced these results.

Future research should focus on longitudinal studies to better understand infection dynamics over time, as well as metagenomic approaches to explore potential viral diversity beyond the commonly screened pathogens. Additionally, studies assessing viral shedding and infectivity in different host species, particularly those outside the psittacine order, could help clarify the role of non-traditional hosts in viral persistence. Finally, improving biosecurity measures in pet trade and aviculture settings, as well as implementing screening protocols before public bird gatherings, could help mitigate the risk of further pathogen spread.

## Figures and Tables

**Table 1:** Count of species positive to BFDV detection.

Scientific Name	Common Name	Count
*Amazona aestiva*	Blue-Fronted Amazon	1
*Anser anser x Anser cygnoides*	Domestic Goose	1
*Ara ararauna*	Blue-and-Yellow Macaw	1
*Aratinga solstitialis*	Sun Conure	1
*Carduelis carduelis*	European Goldfinch	1
*Columba livia*	Rock Dove/Pigeon	1
*Erythrura trichroa*	Blue-Faced Parrotfinch	1
*Gallus gallus*	Chicken	3
*Lonchura striata*	White-Rumped Munia	1
*Melopsittacus undulatus*	Budgerigar	11
*Numila meleagris*	Helmeted Guineafowl	1
*Nymphicus hollandicus*	Cockatiel	3
*Otus scops*	Eurasian Scops Owl	1
*Parabuteo unicinctus*	Harris’s Hawk	2
*Pionites leucogaster*	White-Bellied Caique	2
*Pionites melanocephalus*	Black-Headed Caique	1
*Poephila cincta*	Long-Tailed Finch	1
*Poicephalus senegalus*	Senegal Parrot	1
*Psittacula eupatria*	Alexandrine Parakeet	1
*Psittacus erithacus*	African Grey Parrot	2
*Serinus canaria*	Domestic Canary	6
*Turdus merula*	Common Blackbird	1

**Table 2:** Count of species positive to APV-1 detection.

Species	Common Name	Count
*Agapornis roseicollis*	Peach-Faced Lovebird	1
*Amazona aestiva*	Blue-Fronted Amazon	1
*Gallus gallus*	Chicken	1
*Lonchura striata*	White-Rumped Munia	1
*Melopsittacus undulatus*	Budgerigar	3

## Data Availability

The data presented in this study are available on request from the corresponding author.
